# Duration of food protein‐induced allergic proctocolitis (FPIAP) and the role of intestinal microbiota

**DOI:** 10.1111/pai.70008

**Published:** 2024-12-04

**Authors:** G. N. Vallianatou, N. Douladiris, L. Mageiros, E. Manousakis, V. Zisaki, M. Galani, P. Xepapadaki, S. Taka, N. G. Papadopoulos

**Affiliations:** ^1^ Allergy Department, 2nd Pediatric Clinic National and Kapodistrian University of Athens Athens Greece; ^2^ Department of Information Technology and Biomedical Sciences The American College of Greece Athens Greece; ^3^ Startbio PC Molecular Diagnostics and Biotechnology Services Startbio Athens Greece; ^4^ University of Manchester Manchester UK

**Keywords:** allergic proctocolitis, enterotypes, food protein‐induced allergic proctocolitis, gut microbiome, infants, intestinal microbiota, microbiome maturation, non‐IgE food allergy, rectal bleeding

## Abstract

**Background:**

Food protein‐induced allergic proctocolitis (FPIAP) is the leading cause of rectal bleeding in infants. Tolerance is presumed to develop until the first year of age, although natural history studies are scarce, making the determination of the ideal duration for any intervention, challenging. Intestinal microbiota (IM) is crucial in food allergy development; however, data for FPIAP remain limited. This study aimed to assess FPIAP remission after 3 months of milk avoidance and its correlation with IM longitudinal changes.

**Methods:**

A prospective observational study of infants aged ≤6 months with a diagnosis of FPIAP. After 3 months of management according to a clinical algorithm, infants were subjected to a milk challenge using either a cow (CM) or a goat (GM) milk formula in a random order. Stool samples were collected longitudinally for microbiome analysis.

**Results:**

Out of 61 infants, 57 were challenged (29 with CM, 28 with GM). Of these, 55 (96.5%) achieved tolerance, with no difference in tolerance rates between CM (28/29) and GM (27/28). The average age of tolerance development was 6.3 months. Enterobacteriaceae clusters (Klebsiella‐ and Shigella‐dominated) were most often represented in samples from symptomatic infants. In contrast, Bacteroides and Bifidobacteria clusters emerged later, in apparently healthy infants.

**Conclusion:**

A 3‐month intervention was sufficient for almost all infants to achieve tolerance. GM was tolerated equally well to CM. Symptomatic FPIAP is associated with immature enterotypes, while disease remission coincides with microbiome changes in time.

**Key message**

In the large majority of patients, food protein‐induced allergic proctocolitis lasts for 3 months or less following a successful dietary intervention, in parallel and possibly correlating with intestinal microbiota longitudinal changes. The high rate of early tolerance calls for a review of guidelines and established practices.

## 1 INTRODUCTION

FPIAP is a phenotype of non‐IgE‐mediated food allergy (Non‐IgE FA) that manifests in neonates and young infants with rectal bleeding (RB).^1^, ^2^, ^3^ According to Martin et al., its cumulative incidence rises up to 17%,^4^ while among infants with RB is the most common cause, with a frequency of up to 64%.^1^, ^5^, ^6^, ^7^, ^8^ Clinical guidelines and practice vary significantly,^4^, ^9^, ^10^ while the rarity of clinical studies, gaps in understanding its natural history, and the lack of reliable diagnostic tests^11^, ^12^, ^13^, ^14^, ^15^ largely result in “empirical” management. Severe dietary restrictions on the mother with long‐term avoidance diets of CM, or other foods suspected as antigens,^16^ or/and the use of “hypoallergenic” formulae may negatively impact the family's quality of life and predispose to IgE FA development to offending foods.^4^, ^17^, ^18^, ^19^ The duration of FPIAP treatment, based on CM avoidance, is 6–9 months or until the first year of age when tolerance is presumed,^20^, ^21^, ^22^ although often this period is prolonged.^8^, ^23^, ^24^, ^25^ Nonetheless, the natural history of FPIAP hasn't been studied sufficiently to determine the ideal duration for any intervention.

The interaction between the IM and the innate immune system appears to determine the development and natural history of FA.^26^, ^27^, ^28^, ^29^, ^30^, ^31^ Non‐IgE FA, especially FPIAP, has not been sufficiently studied, despite its onset during a period of radical changes in the IM. A recent study identified dysbiotic features in the IM of children with FPIAP^28^; however, data remain limited.

Herein, we hypothesized that the natural course of FPIAP involves faster resolution, and thus, medical interventions can last less than currently practiced. We also hypothesized that the IM longitudinal changes and specific enterotypes are related to the onset and outcome of the disease. Our aim was to assess the degree of FPIAP remission after 3 months of milk avoidance and its correlation with the degree of IM changes. Additionally, we aimed to evaluate if there are differences in reactivity between infant formulas based on CM or GM.

## 2 MATERIALS AND METHODS

### 2.1 Patients and study design

Infants referred to our Unit between April 2019 and January 2022 due to bloody stools/suspicion of FPIAP were invited to participate. The study was announced to the Unit's network of collaborating pediatricians to increase referrals. Inclusion criteria were a. age ≤6 months, b. report of blood with or without mucus in the stools, and c. an otherwise unremarkable clinical presentation. Exclusion criteria were a. the presence of an anal fissure or b. inability or unwillingness to follow the study procedures. The study has been approved by the “P&A Kyriakou” Children's Hospital Ethics Committee and participation required written informed consent from the parents.

Baseline laboratory testing included stool cultures (Bioprepare Microbiology, Greece) and viruses‐Adenovirus (RIDASCREEN® ADENOVIRUS, r‐biopharm, Germany), Norovirus (RIDASCREEN® Norovirus—third Generation, r‐biopharm, Germany), Rotavirus (RIDASCREEN® ROTAVIRUS, r‐biopharm, Germany), blood counts (ABX Pentra 60 Hematology Blood Analyzer from HORIBA, France), high‐sensitive C‐Reactive Protein (CRP) (Dimension EXL 200, Siemens Germany), Prothrombin Time, INR (Thromborel® S, BCS XP System, Siemens, Germany) skin prick test (SPT's), total IgE and specific IgE to cow's milk (ImmunoCAP, Thermo Scientific, Sweden). Fecal and serum samples were obtained at the time of the first clinical encounter and during follow‐up according to the study design (Table S1). Skin prick tests with a wheal size of > = 3 mm and specific IgE value >0.35 kUA/L were considered positive. Information on weight, height, presence of any functional gastrointestinal disorders (vomiting, regurgitation, colics, constipation, food refusal), atopic eczema or respiratory symptoms were also recorded, to evaluate potential predictors of the outcome.

The diagnosis was confirmed clinically, and infants were managed according to the standardized clinical protocol of the Unit, depending on the feeding regimen of the infant upon presentation (Figures S1 and S2) They were followed up 2 and 4 weeks after inclusion to confirm response to the feeding regimen or step up in the clinical protocol.

Infants were invited for a milk challenge after the completion of a 3‐month avoidance treatment including all dairy, cow and goat, products, using either a cow's milk or a goat's milk formula in a random order to evaluate their reactivity to cow's or goat's milk, respectively. The challenge protocol is described in detail in the online supplementary. In case of a positive challenge, continuation of the feeding regimen and rechallenging after 6 months has been planned.

The study chart is shown in Table S1.

The primary outcome was the proportion of infants able to tolerate a milk formula 3 months after the beginning of a responsive treatment. Secondary outcomes were the proportion of children tolerating goat's milk versus those tolerating cow's milk and differences in microbiome composition between infants tolerating milk versus those who would not.

### 2.2 Microbiome studies

Human feces were collected with the stool collection tubes with DNA stabilizer (Invitek Diagnostics, Germany). The samples were transferred to the laboratory using a cryobox with ice‐pack. Upon arrival, samples were stored at −80°C. Stored samples were sent to Novogene (Cambridge, UK) for processing and 16srRNA sequencing. OTU clustering, taxonomic annotation, and further analysis were performed in our laboratory using standardized or in‐house tools. Methodological details can be found in the online supplement.

## 3 RESULTS

### 3.1 Population characteristics

Sixty‐one infants (30 girls) were included in the study. Demographics and disease characteristics of the population at inclusion are shown in Table 1. At baseline, IgE sensitization to CM was found in only one infant through SPT, while specific immunoglobulin to cow's milk was negative in all infants tested (48/48). At the time of challenge, SPTs of all infants were negative, while specific immunoglobulin to cow's milk was positive in one infant at a low level (0.57 IU/mL).

**TABLE 1:** Characteristics of the study population at inclusion (*Ν* = 61).

Sex (female)	30 (49%)
Age of symptom initiation	3 days—4.5 months (median 9 weeks)
Duration of pregnancy	
Preterm	10 (16%)
Full‐ Term	51 (84%)
Mode of delivery	
Cesarean delivery	39 (64%)
Vaginal delivery	22 (36%)
Atopic Dermatitis	23 (37.7%)
Respiratory symptoms	8 (13.1%)
CM IgE Sensitization	1 (1.6%)
Functional gastrointestinal disorders	
Regurgitation	30 (49%)
Colic	23 (37.7%)
Food refusal	20 (32.8%)
Constipation	9 (14.7%)
Feeding regimen at presentation	
Exclusive breast feeding	39 (64%)
Mixed feeding	15 (24%)
Formula feeding	7 (12%).

### 3.2 Clinical outcome

**FIGURE 1:**
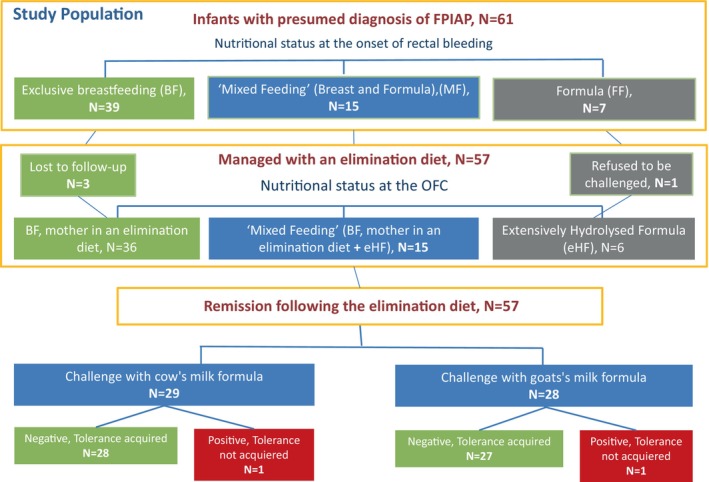
Study population diagram.

Out of 61 infants, 3 were lost to follow‐up and one did not accept to be challenged. In total, 29/36 breast fed infants at the onset of rectal bleeding responded to maternal diet, while 7/36 infants were prescribed extensively hydrolyzed formula (eHF) formula following unresponsiveness to maternal diet. None of the infants were prescribed amino acid‐based formula (AAF). Fifty‐seven infants were challenged after 3 months, 29 with a CM formula and 28 with a GM formula. Of these, 55/57 (96.5%) were tolerant, with no difference in tolerance rates between the CM and GM formulae (28/29 (96.5%), and 27/28 (96.4%), respectively) (Figure 1). The two infants with a positive challenge at 3‐months were re‐challenged 6 months later and were able to tolerate their respective formula.

The average age of tolerance development was 6.3 months (median: 6.2). The average time from the initial occurrence of symptoms was 4.3 months.

Due to the very small proportion of infants who did not develop tolerance, the planned comparisons between tolerant and non‐tolerant infants, as well as the risk prediction analyses were not performed.

### 3.3 Microbiome longitudinal changes

Our dataset contained 114 sequenced samples from 58 patients, taken at three different timepoints. Specifically, we obtained 28 samples at baseline, 35 at midpoint and 51 at endpoint. Nonetheless, we were not able to obtain all 3 timepoints from every patient, as some of them have samples from only 1 or 2 timepoints. Notice that in each of these timepoints we have 23 (out of 28), 2 (out of 35), and 1 (out of 51) samples, respectively, from patients that were shedding blood, (“symptomatic,” Figure 3Β). Details can be found in Table S2.

The 10 most common genera in our samples were Bacteroides, Escherichia‐Shigella, Akkermansia, Bifidobacterium, Clostridium, Blautia, Ruminococcus, Veillonella, Klebsiella, and Lachnoclostridium. The 100 most common genera based on their prevalence in our data can be found in Figure S3.

**FIGURE 2:**
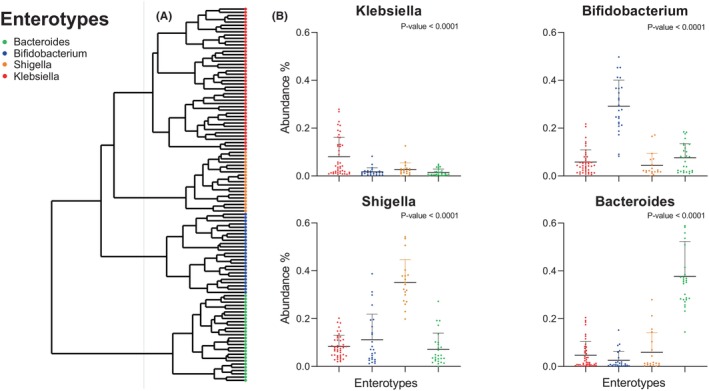
Enterotype clustering and genera prevalence. (A) Four distinct enterotype clusters were identified using hierarchical clustering in our metagenomic data. The gray line indicates the tree point that we used to define our clusters. (B) Each of the clusters contained a predominant bacterial genus, namely Bacteroides (green), Bifidobacterium (blue), Shigella (purple), and Klebsiella (red). Every dot represents a sample, and statistical significance was inferred using a Kruskal–Wallis test.

**FIGURE 3:**
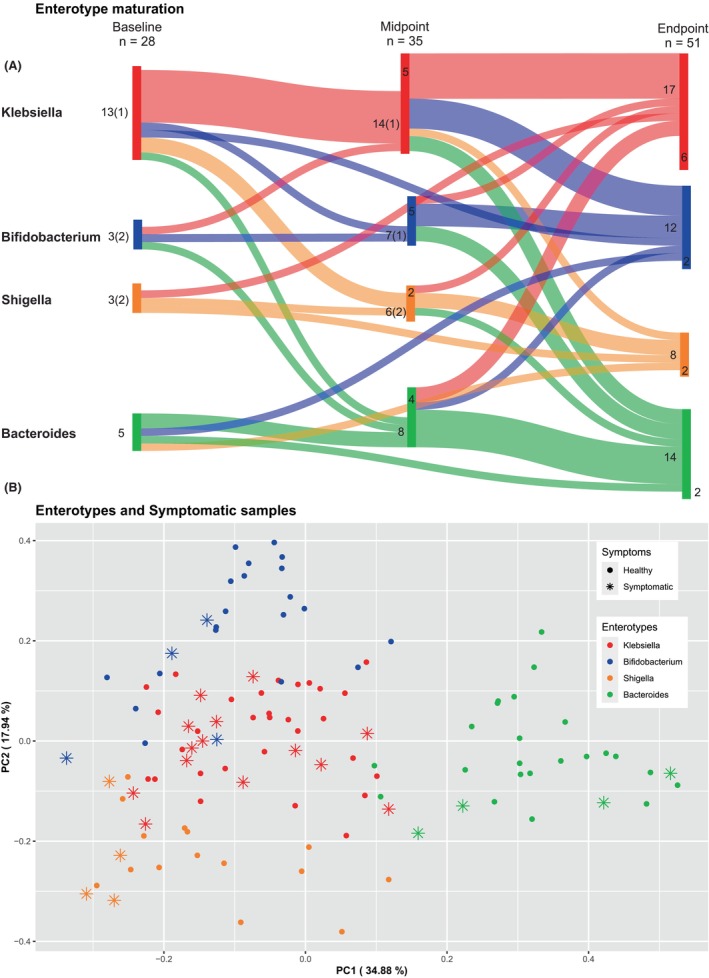
Enterotype longitudinal changes and Principal Coordinate Analysis (PcoA). (A) Sankey plot of the enterotype changes in our cohort within the time frame of the three sampling points. Numbers in parentheses denote the number of samples without a consecutive measurement. The numbers within the bars denote the samples without a previous measurement. (B) PcoA of metagenomic data based on Beta diversity. Enterotype clusters are represented with different colors; symptomatic samples are designated with an asterisk.

Samples were hierarchically clustered based on a pairwise Bray–Curtis dissimilarity matrix at the genus level. Four distinct clusters were revealed, corresponding to previously described enterotypes (Figure 2). The predominant genera (*p*‐value <.001) characterizing these enterotypes were Bacteroides, Bifidobacterium, Shigella, and Klebsiella.^32^, ^33^ Interestingly, the majority of “symptomatic” samples belonged to the Klebsiella (*n* = 14/26, 54%) or the Shigella (*n* = 4/26, 15%) enterotype (Figure 3). Τhe Bifidobacterium and Bacteroides enterotypes appeared in the gut of our participants after the age of 3 months (Figure S4). Bifidobacteria were more abundant in breast fed infants in comparison to those receiving eHF. There was no apparent correlation between the delivery mode of the infants and the corresponding microbiome type, as shown in a PCoA analysis (Figure S5). Finally, observing enterotypes, Bifidobacterium and Bacteroides enterotypes became more prominent in time (Figure 3).

## 4 DISCUSSION

In this study, we have confirmed our hypothesis that the duration of FPIAP is shorter than generally perceived and in the large majority of cases lasts for 3 months of less following a successful dietary intervention, or an average age of about 6 months. This is an important finding, as it has direct consequences in FPIAP management and the development of relevant care pathways. Although the small number of infants with a positive challenge did not allow us to explore potential risk factors or differential microbiome changes in time, as we originally planned, we have also found that symptomatic infants tend to have Klebsiella or Shigella‐dominated microbiomes, which is compatible with an immature microbiome. Furthermore, tolerance is achieved for both cow's and goat's milk with no difference in tolerance rates between the CM and GM formulae. Mammals that are phylogenetically related such as cow and goat have similar milk protein expression.^34^ Significant amino acid sequence homology results in a high rate of clinical cross‐reactivity between CM and GM.^35^, ^36^ Biochemical studies have shown that the casein proteins in CM and GM are quite similar,^37^ evidence that are consistent with the results of our study.

Goat‐based formulae (GMF) have become popular with Greek families, marketed as a “healthier” choice. GMF provides adequate growth, has a good tolerability, and is safe to use in infants.^38^ According to recent data, infants fed with whole milk‐based GMF experienced better food responsiveness and quality of life than those fed with whey‐based CMF, suggesting that whole milk‐based GMF could be an attractive alternative.^39^

In the current management of FPIAP, it is considered that tolerance to milk is achieved at the age of 1‐ ≤2 years depending on the reports.^1^, ^2^, ^3^, ^8^, ^20^, ^21^, ^22^, ^23^, ^24^, ^40^, ^41^ Infants are on an elimination diet for at least 6 months^1^, ^3^, ^22^, ^42^ a time period required—according to current guidelines—before reintroducing full milk^22^, ^40^, ^41^ which is typically done in most patients at the age of 9–12 months.^1^, ^41^, ^42^ In one of the largest series of children with FPIAP, the mean age at which successful reintroduction of cow's milk was performed was 11 months.^4^

However, data regarding the natural history of the disease are ambiguous and contradictory. Some studies suggest that the disease tends to have a more prolonged course in certain cases while other that the disease may be more transient or less frequent.^6^, ^23^, ^43^, ^44^, ^45^, ^46^ Studies suggest that there are factors within the intestinal environment that may cause RΒ without being a response to a specific antigen.^44^, ^47^, ^48^, ^49^ In this context, Idiopathic Neonatal Transient Colitis (INTC) is relevant, an entity that occurs mostly in the first minutes or days after birth.^44^, ^47^, ^48^ Arvola et al. in Finland and Elizur et al. in Israel found that infants with RB on an elimination diet, challenged with CM 1 and 3 months after initial presentation, respectively, showed a lack of symptom reoccurrence in the majority of cases.^6^, ^50^ These studies suggest rechallenging with CM upon resolution of symptoms to better establish the diagnosis. However, as noted by Feuille E et al.^3^ it is not clear if these negative challenges may reflect resolution of FPIAP over these months, rather than misdiagnosis. In line with the above, it is plausible that the duration of the treatment could be shorter. Nevertheless, there have been no sufficient data on the proportion of resolution before the age of 9–12 months; therefore, it is not yet recommended to perform tolerance challenges (OFC‐T) before that age.

Our study supports the notion of a rapid natural history of FPIAP and makes an important step toward defining its duration by determining that the large majority of cases acquire tolerance at an average age of 6.3 months, or less. Our findings align with those of another prospective study by Lemoine et al., where the median age at acquisition of tolerance was 6.8 months.^46^ However, while our study focuses on the main clinical symptom and diagnostic criterion of FPIAP, rectal bleeding, Lemoine's study also takes into account non‐specific subjective symptoms such as significant persistent behavioral changes. In our study, the timing of the OFC‐T was determined by the duration of the responsive treatment that the infants received, rather than their age. Consequently, infants of the study who manifested FPIAP soon after birth confirmed their tolerance as early as at the age of 3 months.

According to recent reports, infants with FPIAP may be at an increased risk for IgE sensitization or IgE‐mediated FA,^17^, ^18^ factors predisposing to a persistent course of the disease.^43^, ^51^ The complete pathogenetic mechanism linking some cases of FPIAP with Th2 immune responses remains to be clarified; however, long‐term exclusion of cow's milk from the diet may play a role. In our study, we observed quite low, almost non‐existent, rates of new sensitization to CM compared to other studies,^17^, ^18^, ^43^ which could be attributed to the short observation window, but also short CM elimination diet.

IM have been shown to play a role from the first days of life^52^ during the neonatal and early infant period as a regulatory factor for shaping the immune system, the development of oral tolerance or the shift toward hypersensitivity reactions when its composition and diversity are disrupted.^26^, ^27^, ^53^, ^54^ Some recent studies correlate the composition of IM with the pathogenesis of allergy^55^ and specifically of FPIAP.^56^ Phylogenetic analysis combined with cultures of the IM in affected and healthy infants by Kumagai et al.^31^ highlighted Klebsiella and Escherichia coli as the dominant strains, respectively. Additionally, Clostridium and Bacteroides strains were detected as dominant in the IM of healthy infants, with rare representation in that of affected infants, reflecting delayed IM maturation in symptomatic infants. Arvola et al.^6^ reported that the levels of Bifidobacterium and Lactobacillus were reduced in FPIAP. Similarly, Nevoral et al.^57^ found significantly lower levels of Bifidobacteria in infants with mucous bloody stools compared to healthy, breastfed infants. Studies suggest that an elimination diet could lead to microbiome longitudinal changes toward health‐related enterotypes.^58^ A recent large microbiome study in infants with FPIAP by Martin et al.^27^ used longitudinal sampling and found higher abundance of Enterobacteriaceae and lower abundance of Clostridiales during the symptomatic period.

Our study also using longitudinal sampling consolidates the above findings and takes a further step in characterizing the microbiota groups involved. We identify enterotypes associated with IM maturity and with health/disease separation: Enterobacteriaceae clusters (specifically Klebsiella and Shigella as dominant genera) linked with immaturity were most often represented in symptomatic samples. In contrast, Bacteroides and Bifidobacteria clusters emerge later, in apparently healthy infants. The absence of Bifidobacteria from the microbiome composition in the first few months of life may contribute to the manifestation of FPIAP, as this “deficiency” negatively affects the physiology, immune responses, and inflammatory responses of the intestinal mucosa.

It is widely recognized that breast feeding induces colonization of the intestinal microbiota by Bifidobacteria contributing to its maturation.^59^, ^60^, ^61^ Despite a small sample, we confirm that Bifidobacteria were more abundant in breastfed infants in comparison with those receiving eHF. In conclusion, observing the transition of enterotypes through our study's sampling points it is apparent that health‐related enterotypes (Bifidobacterium and Bacteroides) tend to grow in numbers as the gut microbiome matures. This supports the possibility for using IM longitudinal phenotype as a potential marker for FPIAP remission, a concept that needs further investigation.

There are some limitations to our study. The number of infants is not high; nevertheless, the very high proportion of infants overcoming FPIAP suggests that the number is sufficient for confirming the hypothesis. The design was observational, based on a clinical algorithm and without a control group. This is fit for a natural history study; furthermore, we consider that any suboptimal intervention would have been unethical for our infants. Equally, the diagnosis was clinical, considering that endoscopic confirmation would not have been acceptable for FPIAP in our population. In line with the rationale of minimal intervention, the choice of conducting only one oral food challenge (OFC) was made. This OFC bridges the gap between diagnostic challenge (OFC‐D) and tolerance challenge (OFC‐T) within the short timeframe of 3 months where tolerance was likely to be detected. Additionally, the study design follows everyday clinical medical practice where diagnostic challenges are rarely carried out.^17^ The main reason for this is parental reluctance to reintroduce CM for diagnostic purposes immediately after the resolution of symptoms, mainly in cases where there have been multiple changes in the breastfeeding mother's diet or various formulae administered to the newborn until remission, or in cases of initial severe symptoms as persistent or significant rectal bleeding. The study was based on a single center, although our catchment area expands over a large part of the country.

Despite these limitations, the findings shed light on the natural history of FPIAP and support the notion of a rapid turnover, in parallel and possibly correlating with intestinal microbiota longitudinal changes. Although a favorable prognosis is already generally accepted for FPIAP, the high rate of tolerance achieved within 3 months of treatment calls for a review of guidelines and established practices and has become the standard of care in our department.

## AUTHOR CONTRIBUTIONS

**G.N. Vallianatou:** Conceptualization; investigation; data curation; writing – original draft; methodology; writing – review and editing; validation; resources; project administration; formal analysis; visualization. **N. Douladiris:** Data curation; resources; validation; methodology. **L. Mageiros:** Software; formal analysis; methodology; visualization; writing – review and editing; writing – original draft; data curation. **E. Manoussakis:** Methodology; validation; data curation; resources. **V. Zisaki:** Resources; data curation. **M. Galani:** Resources; data curation. **P. Xepapadaki:** Data curation; resources. **S. Taka:** Resources; methodology; data curation; formal analysis. **N. G. Papadopoulos:** Conceptualization; writing – original draft; writing – review and editing; data curation; supervision; resources; validation; methodology; funding acquisition; project administration.

## FUNDING INFORMATION

The study was supported by an unrestricted grant by Dairy Goat Co‐operative (DGC). The funder had no influence on the design, interpretation or writing of the study.

## CONFLICT OF INTEREST STATEMENT

N.G.Papadopoulos. Time frame past 36 months. Grants or contracts from any entity: Capricare, Nestle, Numil, Vianex, and REG. Consulting fees: Abbott, Abbvie, Astra Zeneca, GSK, HAL, Medscape, Menarini/Faes Farma, Mylan, Novartis, Nutricia, OM Pharma, and Regeneron/Sanofi. The other authors declare no conflict of interest.

### PEER REVIEW

The peer review history for this article is available at https://www.webofscience.com/api/gateway/wos/peer‐review/10.1111/pai.70008.

## Supporting information


**Figure S1.** Clinical management protocol for infants exclusively breastfeeding or receiving eHF.

**Figure S2.** Clinical management protocol for infants with mixed feeding.

**Figure S3.** Top 100 genera in the metagenomic samples.
The genera selection was made based on the average abundance of the genera in our metagenomic data. Background colors denote different class groups (see legend). The bars in the outer rings indicate the presence of a genus in our samples and every color in the bars corresponds to a specific sample. Every outer ring indicates a 20% interval.

**Figure S4.** Enterotype characterization of distinct age groups.

**Figure S5.** Principal Coordinate Analysis of metagenomic data based on Beta diversity. The birth type of each sample is represented with a different color.

Table S1.

Table S2.

Table S3.

**Appendix S1.** Supporting information.
