# Supplementary material for: Duration of food protein‐induced allergic proctocolitis (FPIAP) and the role of intestinal microbiota
Source: Pediatr Allergy Immunol. 2024 Dec 4;35(12):e70008. doi: 10.1111/pai.70008 (PMC11616471; doi:10.1111/pai.70008)
Supplement: Supplementary file 1 — Figure S1. Clinical management protocol for infants exclusively breastfeeding or receiving eHF. [file PAI-35-e70008-s009.pdf]

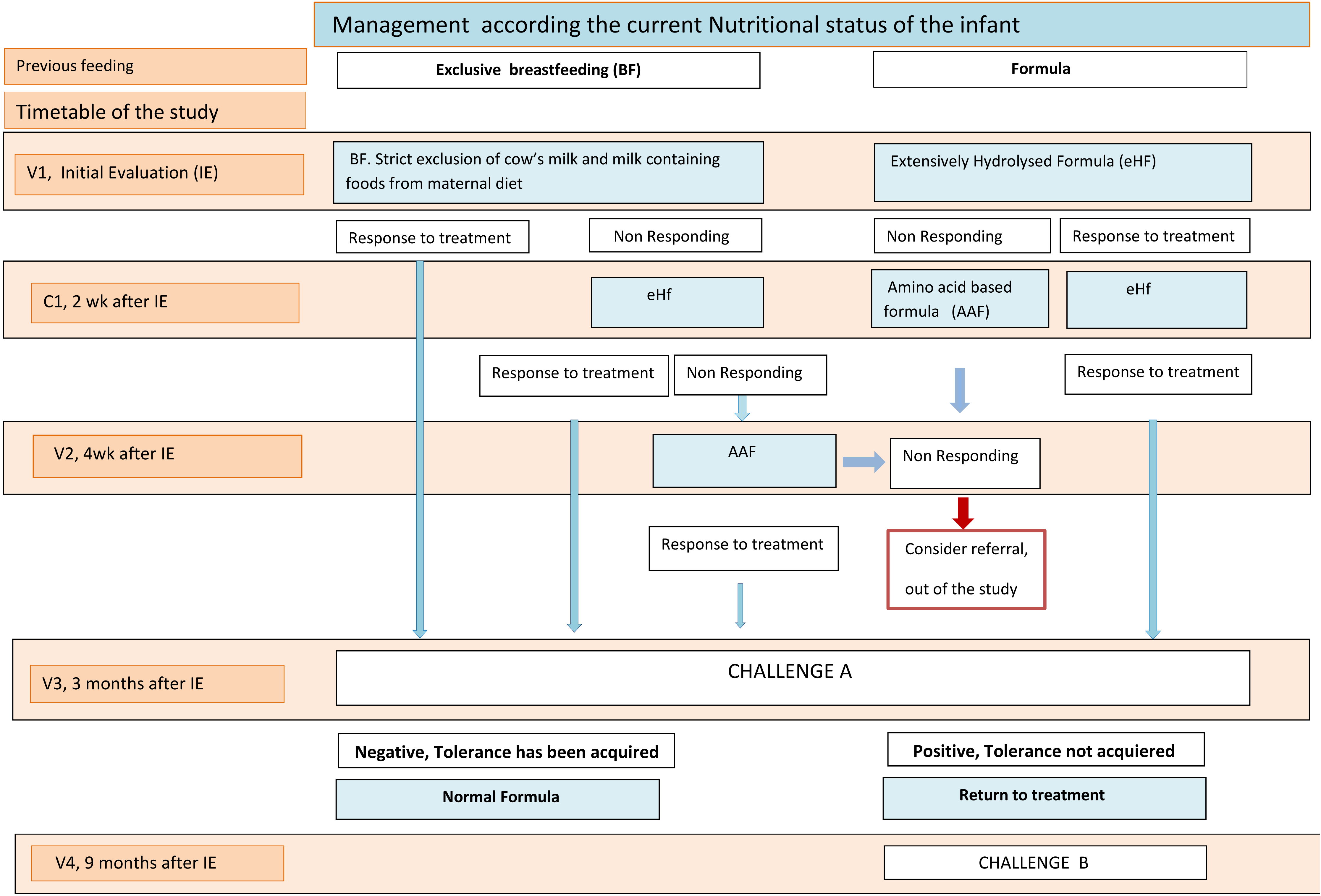

Supplementary Figure 1: Management according the current Nutritional status of the infant. Exclusive breastfeeding or Formula
