# Supplementary material for: Duration of food protein‐induced allergic proctocolitis (FPIAP) and the role of intestinal microbiota
Source: Pediatr Allergy Immunol. 2024 Dec 4;35(12):e70008. doi: 10.1111/pai.70008 (PMC11616471; doi:10.1111/pai.70008)
Supplement: Supplementary file 2 — Figure S2. Clinical management protocol for infants with mixed feeding. [file PAI-35-e70008-s003.pdf]

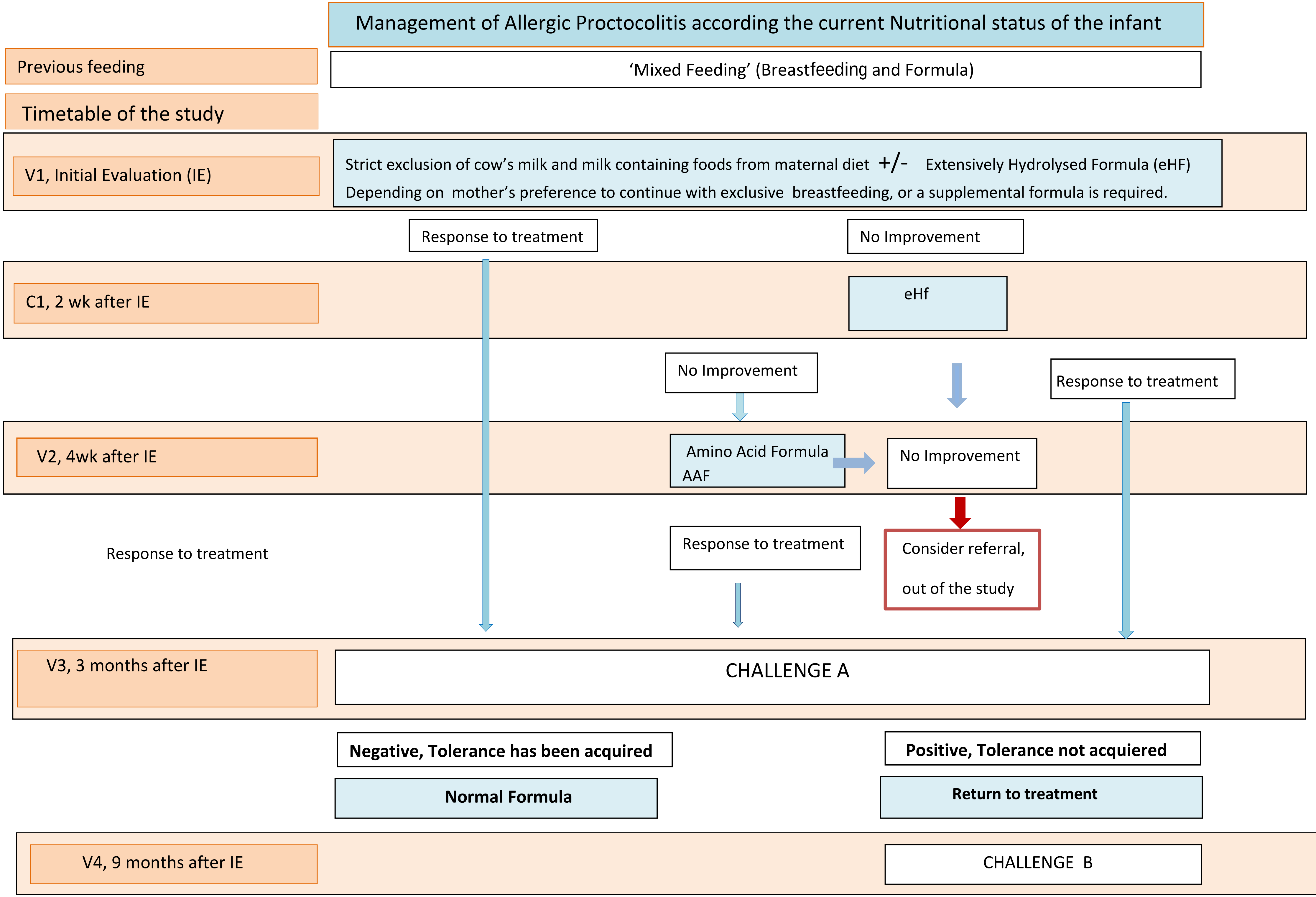

Supplementary Figure 2: Management of Allergic Proctocolitis according the current Nutritional status of the infant. Mixed feeding (Breastfeeding and Formula)
