# Supplementary material for: Duration of food protein‐induced allergic proctocolitis (FPIAP) and the role of intestinal microbiota
Source: Pediatr Allergy Immunol. 2024 Dec 4;35(12):e70008. doi: 10.1111/pai.70008 (PMC11616471; doi:10.1111/pai.70008)
Supplement: Supplementary file 3 — Figure S3. Top 100 genera in the metagenomic samples. The genera selection was made based on the average abundance of the genera in our metagenomic data. Background colors denote different class groups (see legend). The bars in the outer rings indicate the presence of a genus in our samples and every color in the bars corresponds to a specific sample. Every outer ring indicates a 20% interval. [file PAI-35-e70008-s004.pdf]

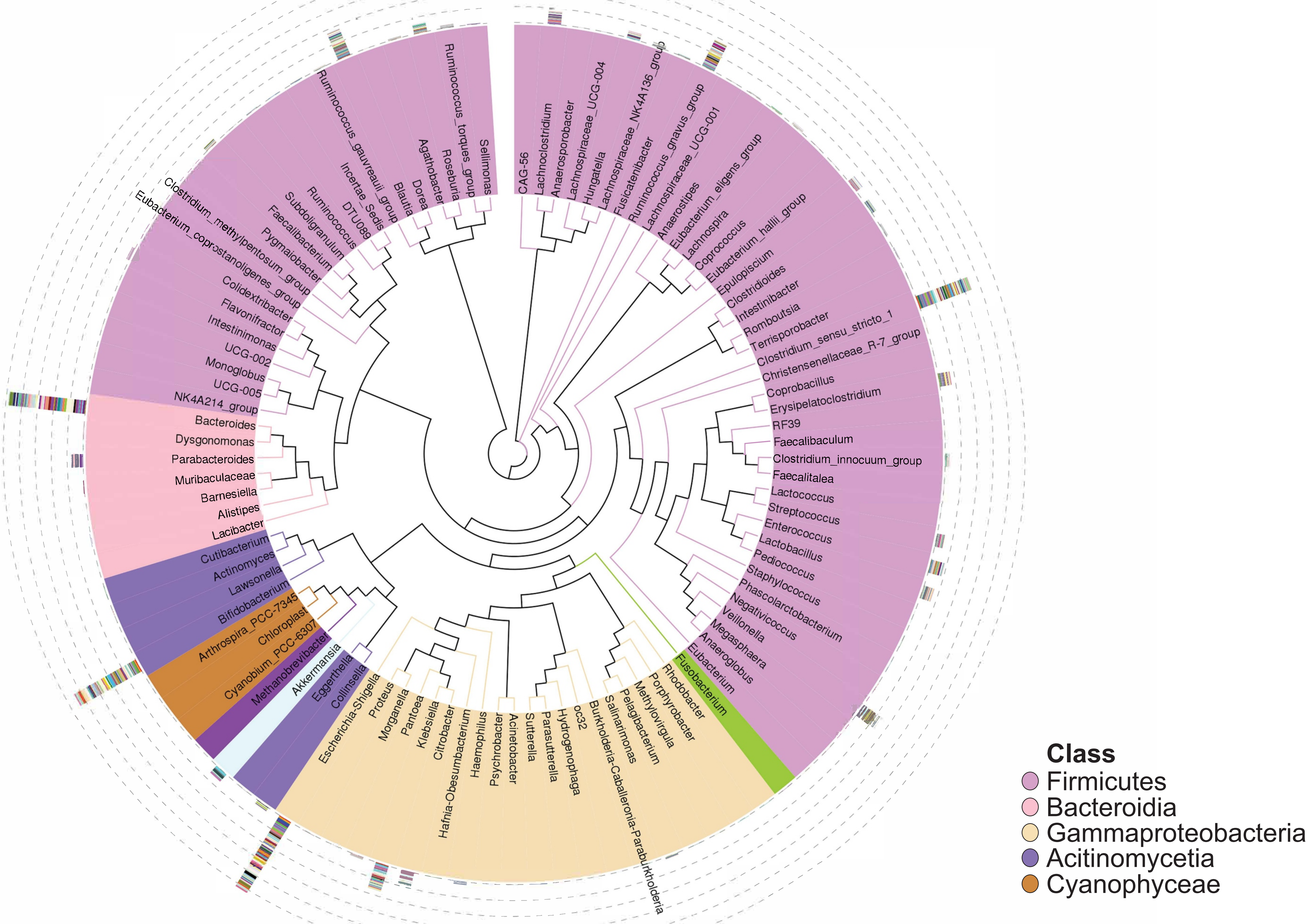


**Supplementary Figure 3: Phylogenetic tree of the 100 most prevalent genera in our metagenomic samples**
