# Supplementary figures and images for: Duration of food protein‐induced allergic proctocolitis (FPIAP) and the role of intestinal microbiota
Source: Pediatr Allergy Immunol. 2024 Dec 4;35(12):e70008. doi: 10.1111/pai.70008 (PMC11616471; doi:10.1111/pai.70008)

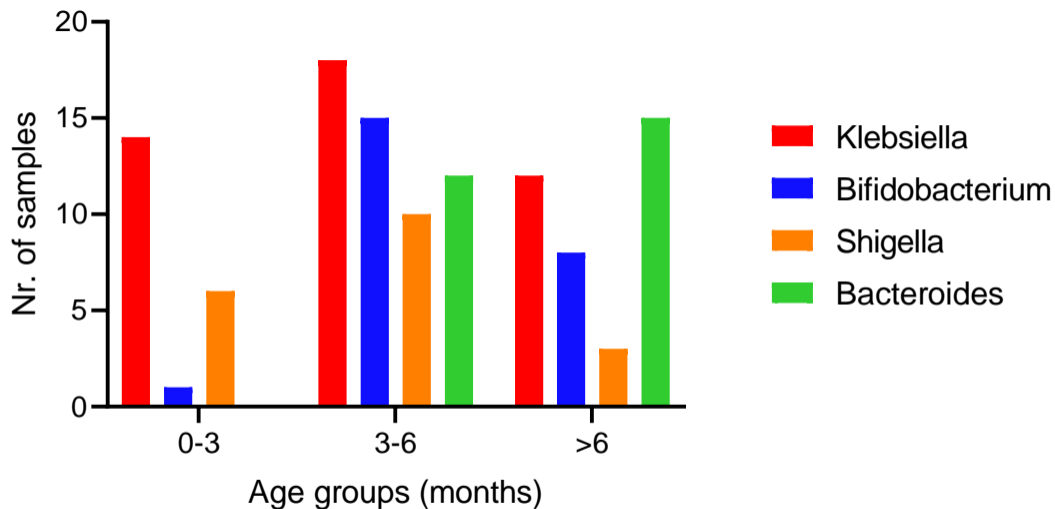

**Supplementary Figure 4: Enterotype characterisation of distinct age groups**

Supplement: Supplementary file 4 — Figure S4. Enterotype characterization of distinct age groups. [file PAI-35-e70008-s005.pdf]
