# Supplementary material for: Duration of food protein‐induced allergic proctocolitis (FPIAP) and the role of intestinal microbiota
Source: Pediatr Allergy Immunol. 2024 Dec 4;35(12):e70008. doi: 10.1111/pai.70008 (PMC11616471; doi:10.1111/pai.70008)
Supplement: Supplementary file 5 — Figure S5. Principal Coordinate Analysis of metagenomic data based on Beta diversity. The birth type of each sample is represented with a different color. [file PAI-35-e70008-s001.pdf]

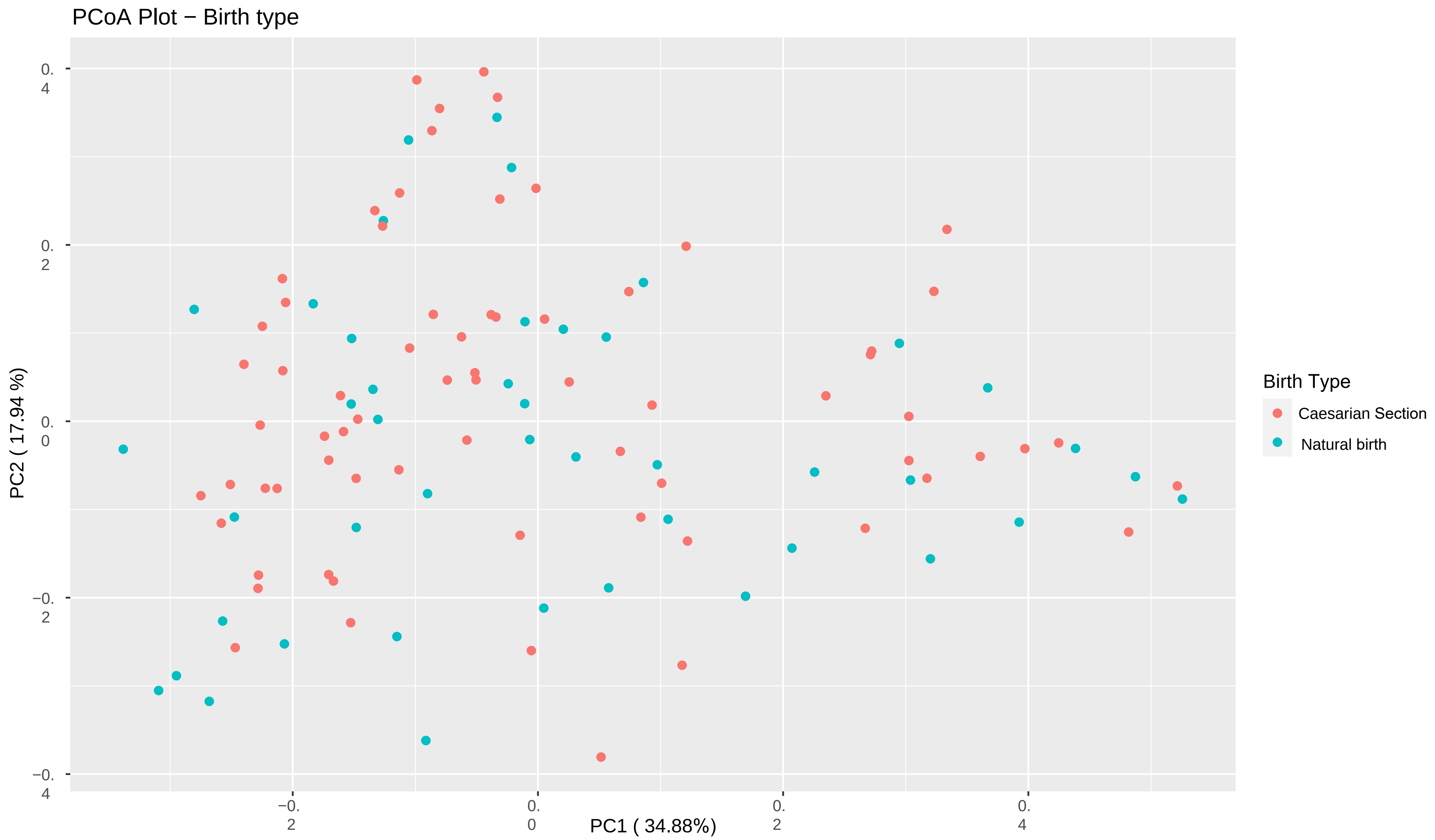

Supplementary Figure 5: Principal Coordinate Analysis of metagenomic data based on Beta diversity. The birth type of each sample is represented with a different color.
