# Supplementary material for: Duration of food protein‐induced allergic proctocolitis (FPIAP) and the role of intestinal microbiota
Source: Pediatr Allergy Immunol. 2024 Dec 4;35(12):e70008. doi: 10.1111/pai.70008 (PMC11616471; doi:10.1111/pai.70008)
Supplement: Supplementary file 6 — Table S1. [file PAI-35-e70008-s008.docx]

**Supplementary Table 1: Study Protocol Table**

|  | V1  **BEGINNING OF STUDY** | C1 | V2 | V3  **CHALLENGE A:** | V4  **CHALLENGE B:**  In case of positive challenge Α | |
| --- | --- | --- | --- | --- | --- | --- |
|  | Unit | Telephone contact | Unit | Unit | Unit | |
| Timetable | 0, Initial Evaluation (IE) | 2 weeks after IE | 4 weeks after IE | 3 months after IE | 9 months after IE | |
| Description | **1.** Evaluation and selection of infants for participation in the study based on clinical criteria.  **2.** Parent receive instructions about:  - treatment plan  - monitoring  **3.** Initial laboratory testing and other tests, as described in detail in the table.  **4.** Parents are briefed on the study and consent form is handed out to them. | **1**.Parent report on:  **a)** response to initial treatment  **b)** weight gain and possible changes in the clinical picture (regurgitation, colic, constipation, food refusal)  **2.** Confirmation that the infant’s participation will continue.  **3.** Treatment adjustment in case of no response | **1.** Treatment response evaluation.  **2.** Laboratory testing as described in detail in the table.  **3.** Confirmation that the infant’s participation will continue.  **4.** Treatment adjustment in case of no response. | **1.** Treatment response evaluation.  **2.** Laboratory testing and other tests, as described in detail in the table.  **3.**Instructions depending on the result:  **a.** In case of a positive challenge: continuation of treatment.  **b.** In case of a negative challenge: instructions for change to a standard milk formula. | | **1**.Instructions depending on the result:  **a.** In case of a positive challenge: continuation of treatment  **b.** In case of a negative challenge: instructions for change to a standard milk formula. |
| Symptom diary completed by the parent |  | **●** | **●** |  | | |
| Informed consent signed by the parent | **●** |  |  |  | | |
| Physical examination, clinical evaluation. | **●** |  | **●** | **●** **●** | | |
| Skin tests (SPT’s) | **●** |  |  | **●** **●** | | |
| Total IgE | **●** |  |  | **●** **●** | | |
| f2 | **●** |  |  | **●** **●** | | |
| Complete blood count | **●** |  |  |  | | |
| CRP | **●** |  |  |  | | |
| Prothrombin Time(PT) /INR | **●** |  |  |  | | |
| Feces culture | **●** |  |  |  | | |
| Rota/Adenovirus test | **●** |  |  |  | | |
| Norovirus test | **●** |  |  |  | | |
| Microbiome Analysis | **●** |  | **●** | **●** **●** | | |
