# Supplementary material for: Duration of food protein‐induced allergic proctocolitis (FPIAP) and the role of intestinal microbiota
Source: Pediatr Allergy Immunol. 2024 Dec 4;35(12):e70008. doi: 10.1111/pai.70008 (PMC11616471; doi:10.1111/pai.70008)
Supplement: Supplementary file 9 — Appendix S1. Supporting information. [file PAI-35-e70008-s007.docx]

**Appendix**

**FPIAP Milk challenges**

Infants were subjected to an open oral milk challenge, according to the Unit’s standardized protocol: at the clinic, following clinical examination and vital sign measurements, infants were provided with 35ml of age-appropriate milk formula, in a stepwise manner (5ml, 10ml, 20ml, at 20’ intervals). The infant was observed for 2 hours after the last dose. Subsequently, the parents were instructed to gradually introduce a full formula meal at home, adding 30ml every day and observing the infant’s stools for the presence of blood and/or mucus. Mothers of fully breast-fed infants were also instructed to freely introduce dairy products into their diet. The observation period was at least one week.

The procedure was identical for either cow’s or goat’s milk products

**DNA isolation**

DNA isolation from feces samples was performed using PSP® Spin Stool DNA Basic Kit (Invitek Diagnostics, Germany).

**Library construction, quality control and sequencing**

Stored samples were sent to Novogene. Briefly, DNA was extracted from the samples, followed by a PCR amplification and purification step. PCR amplification of targeted regions was performed by using specific primers connecting with barcodes. The PCR products with proper size were selected by 2% agarose gel electrophoresis. The same amount of PCR products from each sample was pooled, end-repaired, A-tailed and further ligated with Illumina adapters. Libraries were sequenced on a paired-end Illumina platform to generate 250bp paired-end raw reads. The library was checked with Qubit and real-time PCR for quantification and bioanalyzer for size distribution detection.

**Sequencing data processing**

Paired-end reads were assigned to samples based on their unique barcodes and truncated by cutting off the barcode and primer sequences. Paired-end reads were merged using FLASH (V1.2.7) (see details http://ccb.jhu.edu/software/FLASH/), a very fast and accurate analysis tool, which was designed to merge paired-end reads when at least some of the reads overlap the read generated from the opposite end of the same DNA fragment, and the splicing sequences were called raw tags. Quality filtering on the raw tags were performed under specific filtering conditions to obtain the high-quality clean tags according to the Qiime (V1.7.0) (see details http://qiime.org/scripts/split_libraries_fastq.html) quality-controlled process. The tags were compared with the reference database (SILVA138 database, see details http://www.arb-silva.de/) using UCHIME algorithm (UCHIME Algorithm, see details http://www.drive5.com/usearch/manual/uchime_algo.html) to detect chimera sequences (see details https://drive5.com/usearch/manual/chimeras.html). And then the chimera sequences were removed. Then the Effective Tags were obtained. Quality control statistics are shown in Supplementary Table 3

**OTU cluster and taxonomic annotation**

Sequences analyses were performed by Uparse software (Uparse v7.0.1090, see details <http://drive5.com/uparse/>) using all the effective tags. Sequences with ≥97% similarity were assigned to the same OTUs. Representative sequence for each OTU was screened for further annotation. For each representative sequence, Qiime (Version 1.7.0, see details http://qiime.org/scripts/assign_taxonomy.html) in Mothur method was performed against the SSUrRNA database of SILVA138 Database (see details http://www.arb-silva.de/)for species annotation at each taxonomic rank (Threshold:0.8~1) (kingdom, phylum, class, order, family, genus, species). To obtain the phylogenetic relationship of all OTUs representative sequences, the MUSCLE (Version 3.8.31, see details http://www.drive5.com/muscle/) can compare multiple sequences rapidly. OTUs abundance information were normalized using the sample with the least sequences. Subsequent bioinformatics analysis was performed based on the normalized data.

**Bioinformatic analyses**

Downstream bioinformatics analysis was performed in R (4.3.0). From the OTU table we removed low abundant genera (average abundance in all samples < 0.01%). To identify the optimal number of clusters K for our dataset we used a K-means Elbow Method analysis for different values of K. We then performed Hierarchical clustering with K=4, using the Bray-Curtis dissimilarity matrix of all our samples (vegan package). Enterotypes were assigned to each cluster by comparing the relative abundance of the most predominant genera. Statistical significance and the corresponding plots were inferred using a Kruskal-Wallis test. We performed a Fisher's exact test to identify statistically significant differences in the infants challenged with either cow or goat milk. Statistical tests where preformed using GraphPad Prism (8.4.2). ). Additionally, we created the Sankey plot using the online tool SankeyMATIC (https://sankeymatic.com). Finally we performed the PCoA analysis and plotting using an in-house developed R script. The key packages used for that were vegan, tidyverse and ggplot2.

FIGURE LEGENDS

Supplementary Figure 1: Clinical management protocol for infants exclusively breastfeeding or receiving eHF

Supplementary Figure 2: Clinical management protocol for infants with mixed feeding

Supplementary Figure 3: Top 100 genera in the metagenomic samples

The genera selection was made based on the average abundance of the genera in our metagenomic data. Background colors denote diﬀerent class groups (see legend). The bars in the outer rings indicate the presence of a genus in our samples and every color in the bars corresponds to a speciﬁc sample. Every outer ring indicates a 20% interval.

Supplementary Figure 4: Enterotype characterisation of distinct age groups

Supplementary Figure 5: Principal Coordinate Analysis of metagenomic data based on Beta diversity. The birth type of each sample is represented with a diﬀerent color.
